# Inhibition of transforming growth factor **α** (TGF-**α**)-mediated growth effects in ovarian cancer cell lines by a tyrosine kinase inhibitor ZM 252868

**DOI:** 10.1038/sj.bjc.6690175

**Published:** 1999-03

**Authors:** B J B Simpson, J M S Bartlett, K G Macleod, G Rabiasz, E P Miller, A L Rae, P Gordge, R E Leake, W R Miller, J Smyth, S P Langdon

**Affiliations:** Imperial Cancer Research Fund Medical Oncology Unit, Western General Hospitals NHS Trust, Edinburgh, EH4 2XU, UK; Department of Surgery, Glasgow Royal Infirmary, Glasgow, G31 2ER, UK; Department of Biochemistry, Glasgow University, Glasgow, G12 8QQ, UK

**Keywords:** tyrosine kinase inhibitor, ovarian cancer, epidermal growth factor receptor, ZM 252868

## Abstract

The modulating effects of the epidermal growth factor (EGF) receptor-specific tyrosine kinase inhibitor ZM 252868 on cell growth and signalling have been evaluated in four ovarian carcinoma cell lines PE01, PE04, SKOV-3 and PE01^CDDP^. Transforming growth factor α (TGF-α)-stimulated growth was completely inhibited by concentrations ≥ 0.3 μM in the PE01 and PE04 cell lines and by ≥ 0.1 μM in SKOV-3 cells. TGF-α inhibition of PE01^CDDP^ growth was reversed by concentrations ≥ 0.1 μM ZM 252868. TGF-α-stimulated tyrosine phosphorylation of both the EGF receptor and c-erbB2 receptor in all four cell lines. The inhibitor ZM 252868, at concentrations ≥ 0.3 μM, completely inhibited TGF-α-stimulated tyrosine phosphorylation of the EGF receptor and reduced phosphorylation of the c-erbB2 protein. EGF-activated EGF receptor tyrosine kinase activity was completely inhibited by 3 μM ZM 252868 in PE01, SKOV-3 and PE01^CDDP^ cells. These data indicate that the EGF receptor-targeted TK inhibitor ZM 252868 can inhibit growth of ovarian carcinoma cells in vitro consistent with inhibition of tyrosine phosphorylation at the EGF receptor. © 1999 Cancer Research Campaign


*British Journal of Cancer* (1999) **79**(7/8), 1098–1103
© 1999 Cancer Research Campaign
Article no. bjoc.1998.0175

### Inhibition of transforming growth factor α (TGF-α)-mediated growth effects in ovarian cancer cell lines by a tyrosine kinase inhibitor ZM 252868

**BJB Simpson[1], JMS Bartlett[2], KG Macleod[1], G Rabiasz[1], EP Miller[1], AL Rae[2], P Gordge[1], RE Leake[3], WR Miller[1], J Smyth[1] and SP Langdon[1]**

[1]Imperial Cancer Research Fund Medical Oncology Unit, Western General Hospitals NHS Trust, Edinburgh EH4 2XU, UK; [2]Department of Surgery, Queen Elizabeth Building, Glasgow Royal Infirmary, Glasgow G31 2ER, UK; [3]Department of Biochemistry, Glasgow University, Glasgow G12 8QQ, UK

**Summary** The modulating effects of the epidermal growth factor (EGF) receptor-specific tyrosine kinase inhibitor ZM 252868 on cell growth and signalling have been evaluated in four ovarian carcinoma cell lines PE01, PE04, SKOV-3 and $PE01^{CDDP}$. Transforming growth factor α (TGF-α)-stimulated growth was completely inhibited by concentrations ≥ 0.3 μM in the PE01 and PE04 cell lines and by ≥ 0.1 μM in SKOV-3 cells. TGF-α inhibition of $PE01^{CDDP}$ growth was reversed by concentrations ≥ 0.1 μM ZM 252868. TGF-α-stimulated tyrosine phosphorylation of both the EGF receptor and c-erbB2 receptor in all four cell lines. The inhibitor ZM 252868, at concentrations ≥ 0.3 μM, completely inhibited TGF-α-stimulated tyrosine phosphorylation of the EGF receptor and reduced phosphorylation of the c-erbB2 protein. EGF-activated EGF receptor tyrosine kinase activity was completely inhibited by 3 μM ZM 252868 in PE01, SKOV-3 and $PE01^{CDDP}$ cells. These data indicate that the EGF receptor-targeted TK inhibitor ZM 252868 can inhibit growth of ovarian carcinoma cells in vitro consistent with inhibition of tyrosine phosphorylation at the EGF receptor.

**Keywords:** tyrosine kinase inhibitor; ovarian cancer; epidermal growth factor receptor; ZM 252868

Protein tyrosine kinases are mediators of growth factor-induced cell proliferation in many cancers, and the type I receptor tyrosine kinase (RTK-I) family of receptors have particularly important roles in ovarian cancer (Simpson et al, 1995). The first identified member of this family, the epidermal growth factor (EGF) receptor (c-erbB-1), is a transmembrane glycoprotein that mediates the mitogenic response to the EGF family of ligands that includes both EGF and transforming growth factor α (TGF-α) (Carpenter, 1987). On activation, the EGF receptor phosphorylates tyrosine residues on its C-terminal tail and may also interact with other members of the RTK-I family (c-erbB2, c-erbB3 and c-erbB4). These intracellular phosphorylations initiate signalling cascades which eventually result in gene activation (Egan and Weinberg, 1993).

The EGF receptor is reported to be present in between 33% and 75% of ovarian cancers (Bauknecht et al, 1988, 1993; Battaglia et al, 1989; Berchuck et al, 1991; Morishige et al, 1991; Owens et al, 1991; Henzen-Logmans et al, 1992), and has been implicated in both the growth and progression of this disease. Ovarian adenocarcinomas that express increased concentrations of the EGF receptor are associated with poor survival (Bauknecht et al, 1988; Battaglia et al, 1989; Foekens et al, 1990; Berchuck et al, 1991; Scambia et al, 1992; Bartlett et al, 1996), and both TGF-α and EGF have been shown to stimulate growth of ovarian cancer cells in culture (Morishige et al, 1991; Rodriguez et al, 1991; Scambia et al, 1991; Crew et al, 1992; Zhou and Leung, 1992).

*Received 9 March 1998*
*Revised 18 June 1998*
*Accepted 25 June 1998*

*Correspondence to:* SP Langdon, ICRF Medical Oncology Unit, Western General Hospital, Edinburgh EH4 2XU, UK

Enzymatic activity of the intracellular tyrosine kinase domain of the EGF receptor is essential for signal transduction (Chen et al, 1987; Honegger et al, 1987). These signalling pathways that mediate cell proliferation therefore represent novel target sites for anti-cancer drug development. The potential use of protein tyrosine kinase inhibitors as antiproliferative agents was first proposed in 1981 for quercetin (Graziani et al, 1981). Recently, many more specific and potent inhibitors have been identified (Fry et al, 1994*a*; Levitzki and Gazit, 1995), and one of these, ZM 252868 [PD 153035; 4(3-bromoanilino)-6,7-dimethoxyquinazoline], is a potent inhibitor of the EGF receptor (Fry et al, 1994*b*; Wakeling et al, 1996; Jones et al, 1997). In this study, we have assessed the ability of the drug to inhibit ovarian cancer cell growth and tyrosine phosphorylation on the EGF receptor in order to obtain evidence that ovarian cancer might be one of the disease types which could be amenable to an EGF receptor-targeted strategy.

### MATERIALS AND METHODS

### Cell lines

The human ovarian carcinoma cell lines PE01 and PE04 were established and characterized as previously described (Langdon et al, 1988). The $PE01^{CDDP}$ variant was established by in vitro exposure of the PE01 line to cisplatin (Beattie et al, 1993). The SKOV-3 ovarian carcinoma cell line was obtained from the European Collection of Animal Cell Cultures, Porton Down, UK. All these lines were routinely cultured at 37°C in an atmosphere of 5% carbon dioxide/95% air in RPMI-1640 containing phenol red indicator. The medium was supplemented with 10% fetal calf serum (FCS), L-glutamine (2 mM), penicillin (100 IU $ml^{-1}$) and streptomycin (100 μg $ml^{-1}$).

### Growth assays

Log-phase PE01, PE01^CDDP^, PE04 and SKOV-3 ovarian cancer cells were harvested by trypsinization and seeded in 24-well plates (Falcon) at a density of $2.5 \times 10^4$ cells per well, or in the case of the SKOV-3 cells at $1.25 \times 10^4$ cells per well, in quadruplicate in RPMI-1640 (Gibco BRL, Paisley, UK) containing 10% heat-inactivated FCS and penicillin (100 U $ml^{-1}$) and streptomycin (100 µg $ml^{-1}$). Cells were maintained routinely at 37°C in a humidified atmosphere of 5% carbon dioxide in air. After 24 h, the media were removed and, after two phosphate-buffered saline (PBS) washes, replaced by phenol-red-free RPMI-1640 containing 5% double charcoal-stripped fetal calf serum (DCS FCS), penicillin (100 U $ml^{-1}$), streptomycin (100 µg $ml^{-1}$) and glutamine (2 mmol $l^{-1}$). Phenol-red-free medium was used because the PE01 and PE04 are growth-stimulated by oestrogen, and the absence of the oestrogenic indicator provides a cleaner system in which to observe TGF-α modulations. After a further 24 h, media were removed and replenished by fresh phenol-red-free RPMI plus additives. Phenol-red-free medium was used because the PE01 and PE04 cell lines are growth stimulated by oestrogen and phenol red has oestrogenic activity. The tyrosine kinase inhibitor ZM 252868 was added 30 min before the addition of TGF-α ($10^{-10}$ M); this time point was designated day 0. Thereafter, fresh medium was added on day 2. Cells were harvested on day 5 and counted using a Coulter counter (Coulter Electronics, Luton, UK).

### Phosphotyrosine Western blotting

PE01, PE01^CDDP^, PE04 and SKOV-3 ovarian cancer cells were grown to 80% confluence in 25 $cm^2$ flasks (Falcon) in the presence of RPMI-1640 containing 10% FCS. After two washes with PBS, phenol-red-free RPMI-1640 containing 5% DCS FCS in the presence or absence of ZM 252868 (0.03–3.0 µM) was added, and the cells were incubated overnight at 37°C in a humidified atmosphere of 5% carbon dioxide in air. Cells were then incubated for 30 min in fresh RPMI ± ZM 252868. RPMI ± ZM 252868 was replenished and incubated for a further 5 min in the presence or absence of TGF-α ($10^{-10}$ M). Cells were then washed twice in ice cold PBS, lysed, detached from the flasks and spun at 15 000 r.p.m. at 4°C. Protein content of the resulting supernatant was determined by Bradford assay (Bradford, 1976) and stored at –80°C before phosphotyrosine Western blotting. Protein samples were denatured at 95°C for 5 min in buffer containing sodium dodecyl sulphate (SDS) and mercaptoethanol, then 75 µg of protein was loaded into each lane on a 7.5% polyacrylamide gel. After polyacrylamide gel electrophoresis, proteins were transferred to Immobilon-P polyvinylidene fluoride (PVDF) membrane using a wet transfer system (BioRad Trans Blot Cell). The transfer was carried out overnight at 4°C with 30 V followed by 60 V for 2 h. Proteins were detected using a specific anti-phosphotyrosine mouse monoclonal antibody, p-Tyr (PY20) (Santa Cruz Biotechnology), in conjunction with a chemiluminescence Western Blotting Kit (Boehringer Mannheim). Membranes were blocked in 1% blocking reagent (supplied with kit and containing 10% purified casein protein in maleic acid) diluted in TBS (Tris-buffered saline pH 7.5) for 1 h at room temperature. The membrane was incubated with primary antibody (PY20) diluted to 1 µg $ml^{-1}$ in 0.5% blocking reagent in TBS overnight at 4°C, washed three times for 5 min in TBS-T (TBS with 0.1% Tween 20), twice for 5 min in TBS, incubated twice for 5 min in 0.5% blocking agent in TBS and treated with secondary antibody for 1 h at room temperature. The secondary antibody (anti-mouse IgG-POD/anti-rabbit IgG-POD) was diluted in 0.5% blocking solution in TBS to 160 mU $ml^{-1}$. Finally, the membrane was washed once again, three 5-min washes in TBS-T, then three 5-min washes in TBS. After incubation in luminescence substrate solution, light emission was detected on radiographic film.

### Epidermal growth factor receptor tyrosine kinase enzyme assay

Epidermal growth factor receptor tyrosine kinase enzyme activity was measured in the ovarian cancer cell lines using a Biotrak assay (Amersham, UK). Briefly, membrane preparations were obtained from cells grown to 70% confluence in 175 $cm^2$ flasks. Lysis buffer (50 mM Tris, 1 mM magnesium chloride, 2 mM EDTA, 20 µg $ml^{-1}$ soybean trypsin inhibitor, 50 µg $ml^{-1}$ PMSF) was added (7 ml per 175 $cm^2$ flask) to cell monolayers. Lysates were homogenized and centrifuged at 3000 r.p.m. for 10 min and supernatants then centrifuged at 100 000 ***g*** for 30 min to obtain membrane preparations. These were resuspended in solubilization buffer [50 mM Hepes, 20% glycerol, 1% Triton-X, 0.1% bovine serum albumin (BSA), 0.05% sodium azide] and stored at –80°C until assayed.

Assay reactions included membrane preparation (10 µl), EGF (5 µl; $1.5 \times 10^{-6}$ M), substrate peptide solution (10 µl; peptide 1.5 mM, 135 mM Hepes, 300 µM sodium orthovanadate, 3 mM dithiothreitol, 0.15% Triton X-100, 6% glycerol and 0.05% sodium azide pH 7.4) and magnesium [γ-$^{32}$P]ATP buffer (5 µl; 9.25 kBq, 0.25 µCi per tube). The reaction was initiated by addition of magnesium [γ-$^{32}$P]ATP buffer and allowed to proceed for 30 min. The reaction was terminated by addition of 300 mM orthophosphoric acid (10 µl) containing carmosine. The terminated reaction mixture 30 µl was then pipetted onto a paper disc which was washed with 1% acetic acid (250 ml) and water. The disc was placed into a scintillation vial and counted in a Packard 1900 CA Tri-Car scintillation analyser.

In the presence of enzyme sample and EGF, the $^{32}$P counted on the papers is the sum of non-specific [$^{32}$P]ATP binding, specific binding of phosphorylated peptide and binding of phosphorylated proteins in the cell extract (A). In the absence of EGF and presence of enzyme, the $^{32}$P counted on the papers is the sum of non-specific [$^{32}$P]ATP binding and non-EGF-dependent tyrosine kinase phosphorylation of the peptide and cell extract proteins (B). EGF-dependent tyrosine kinase activity is, therefore, obtained from (A – B).

### Determination of EGF-R, c-erbB2 and c-erbB3 by immunofluorescence

The presence of EGF receptor, c-erbB2 and c-erbB3 proteins was identified on PE01, PE01^CDDP^, PE04 and SKOV-3 cells by immunofluorescence using a flow cytometer. The following antibodies were used: EGF receptor, clone EGFR1 (ICRF, Clare Hall, London UK); c-erbB2, clone CB11 (Novocastra); and c-erbB3, clone RTJ1 (Novocastra). Cells were harvested by trypsinization (in pilot experiments found to be less damaging than cell scraping), washed in cold PBS containing 5% FCS, and aliquots of approximately $10^6$ cells were then incubated for 60 min with antibody. For c-erbB2 and c-erbB3 staining, 1% saponin (BDH, Poole, Dorset, UK) was added to the cells before antibody addition (Brotherick et al, 1995). Cells were then washed in PBS/FCS and incubated with sheep anti-mouse fluorescein isothiocyanate (FITC, 1:20) for 60 min and washed twice with PBS/FCS. Cells

© Cancer Research Campaign 1999

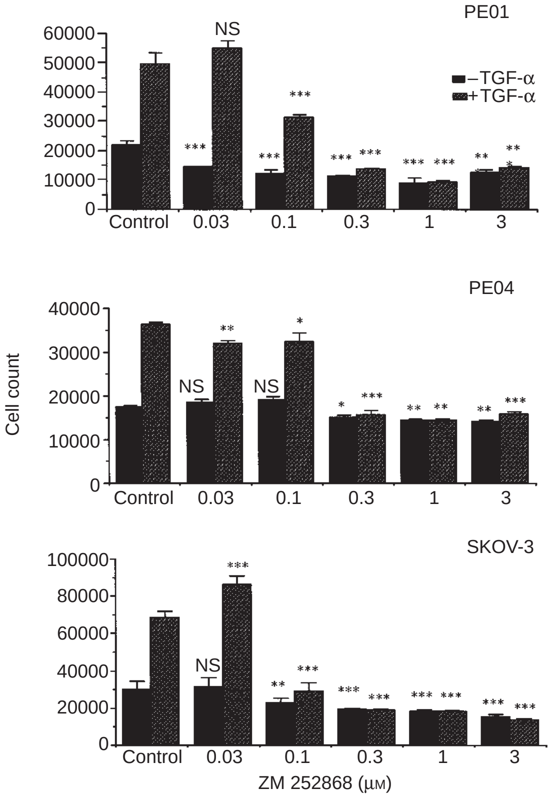

**Figure 1** Effect of ZM 252868 on the basal and TGF-α-stimulated growth of the PE01, PE04 and SKOV-3 ovarian cancer cell lines. Inhibitor was added in the absence (■) or presence of TGF-α ($10^{-10}$ M) (▩). Significantly different from appropriate control: ***$P$ < 0.001; **$P$ < 0.01; *$P$ < 0.05; NS, not significant (Student's *t*-test)

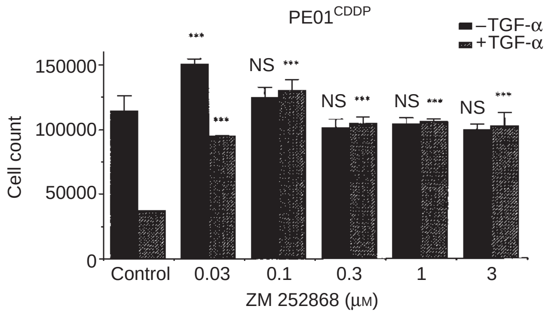

**Figure 2** Effect of ZM 252868 on the basal and TGF-α-inhibited growth of PE01CDDP ovarian cancer cells. Inhibitor was added in the absence (■) or presence of TGF-α ($10^{-10}$ M) (▩). Significantly different from appropriate control: ***$P$ < 0.001; **$P$ < 0.01; *$P$ < 0.05; NS, not significant (Student's *t*-test)

were resuspended in PBS and analysed on the FACScan flow cytometer.

### RESULTS

### Effects of tyrosine kinase inhibitor on TGF-α-modulated growth of ovarian cancer cells

The PE01, PE04, SKOV-3 and PE01CDDP cell lines were treated with ZM 252868 for 5 days in the absence or presence of TGF-α ($10^{-10}$ M) (Figures 1 and 2). In the absence of TGF-α, ZM 252868 inhibited growth of PE01 cells at concentrations equal to or greater than 0.03 μM. Addition of TGF-α produced an approximately 150% increase in control cell number; this increase was abolished by ZM 252868 at concentrations equal to or greater than 0.3 μM (Figure 1A). For PE04 cells, ZM 252868 inhibited growth at 0.3 μM (Figure 1B). Addition of TGF-α produced a 100% increase in cell number and this stimulation was partially reversed at 0.03 μM and completely blocked at 0.3 μM (Figure 1B). For SKOV-3 cells, ZM 252868 inhibited growth at 0.1 μM. Addition of TGF-α produced an approximately 130% increase in control cell number; this increase was abolished by ZM 252868 at concentrations equal to or greater than 0.1 μM (Figure 1C).

Addition of TGF-α to PE01CDDP cells produces growth inhibition (Figure 2). ZM252868 (0.03–3.0 μM) reversed the TGF-α-induced growth inhibition, with a total reversal of this effect observed at concentrations ≥ 0.1 μM. Basal cell growth was also significantly increased ($P$ < 0.001) after the addition of ZM 252868 at 0.03 μM, but was not significantly different from control at concentrations ≥ 0.1 μM (Figure 2).

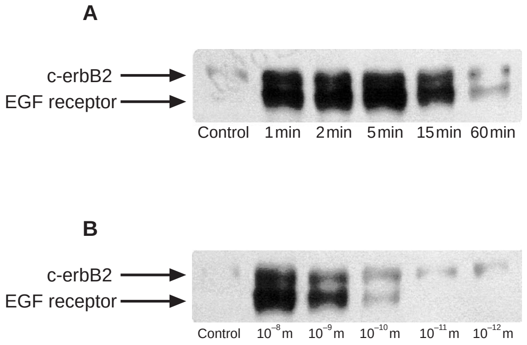

**Figure 3** Western blotting of phosphotyrosine residues in PE01 cells using the PY20 antibody after addition of TGF-α. (**A**) Time course of TGF-α activation in PE01 cells. (**B**) Effect of TGF-α concentration on EGF receptor activation in PE01 cells (after 5 min). The upper signal shown corresponds to the c-erbB protein and the lower signal to the EGF receptor. In untreated cells, only c-erbB2 is tyrosine phosphorylated, but after addition of TGF-α phosphotyrosine signals are seen on the EGF receptor and are increased on c-erbB2 compared with no treatment

### Activation of EGF receptor and c-erbB2 by TGF-α

Western blotting with an anti-phosphotyrosine antibody (PY20) showed that, after exposure to TGF-α, both the EGF receptor and c-erbB2 protein were phosphorylated on tyrosine residues in PE01 cells. The identity of bands was confirmed in parallel experiments using antibodies specific for the EGF receptor and c-erbB2 (data not shown). The time course of activation by TGF-α is illustrated in Figure 3 for the PE01 cell line and occurred within 1 min of addition of the growth factor (Figure 3A). Investigation of the concentration range $10^{-12}$–$10^{-8}$ M indicated that phosphorylation increased with increasing concentrations of TGF-α after a 5-min exposure (Figure 3B). This latter concentration ($10^{-8}$ M) was selected for experiments with ZM 252868.

### Inhibition of tyrosine phosphorylation of the EGF receptor by ZM 252868

The cell lines were treated with ZM 252968 for 5 min in the presence or absence of $10^{-8}$ M TGF-α. TGF-α increased tyrosine

 *© Cancer Research Campaign 1999*

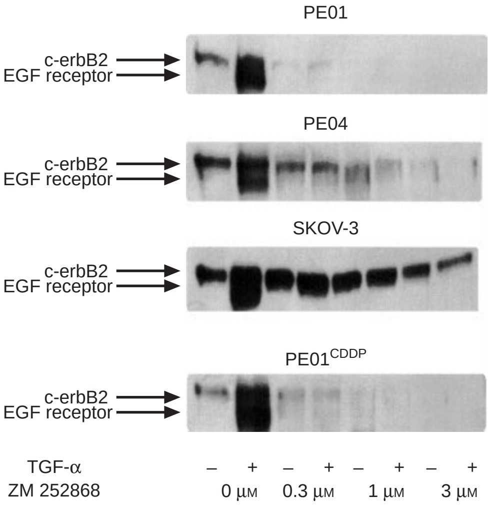

**Figure 4** Western blotting of phosphotyrosine in ZM 252868-treated ovarian cancer cells. Blots are shown for the PE01, PE04, SKOV-3 and PE01$^{CDDP}$ cell lines. The upper signal shown corresponds to the c-erbB2 protein and the lower signal to the EGF receptor. In untreated cells, only c-erbB2 is tyrosine phosphorylated, but after addition of TGF-α phosphotyrosine signals are seen on the EGF receptor and are increased on c-erbB2 compared with no treatment. ZM 252868 was added 30 min before TGF-α addition. Lysates were collected after 5 min exposure to TGF-α. At the concentrations shown, ZM 252868 eliminated the EGF phosphotyrosine signal associated with the EGF receptor and reduced the signal associated with c-erbB2

**Table 1** ZM 252868 inhibition of EGF receptor tyrosine kinase activity in ovarian cancer cell lines

| Cell line | Tyrosine kinase activity (pmol phosphate min$^{-1}$) –EGF Control | +EGF (0.5 μM) | +EGF (0.5 μM) +ZM 252868 (3 μM) |
|---|---|---|---|
| PE01 | 5.93 ± 0.16 | 7.22* ± 0.38 | 4.92 ± 0.41 |
| PE01$^{CDDP}$ | 6.24 ± 0.23 | 8.78* ± 0.50 | 5.67 ± 0.07 |
| SKOV-3 | 3.56 ± 0.11 | 5.32* ± 0.45 | 3.82 ± 0.15 |
| PE04 | 5.44 ± 0.52 | 5.92 ± 0.33 | 5.31 ± 0.89 |

Values shown are means ± standard errors of 4–6 replicates in a typical experiment. *Significantly different from control $P < 0.05$ (Student's *t*-test).

**Table 2** Effect of varying concentrations of ZM 252868 on EGF receptor tyrosine kinase activity in PE01 cells

| EGF (μM) | ZM 252868 (μM) | Activity (pmol phosphate min$^{-1}$) |
|---|---|---|
| 0 | 0 | 3.57 ± 0.1 |
| 0.5 | 0 | 4.74* ± 0.35 |
| 0.5 | 0.03 | 3.64 ± 0.06 |
| 0.5 | 0.3 | 3.59 ± 0.12 |
| 0.5 | 3.0 | 3.40 ± 0.17 |

Values shown are means ± standard error of eight replicate values. *Significantly different from control ($P$ = 0.006, Student's *t*-test).

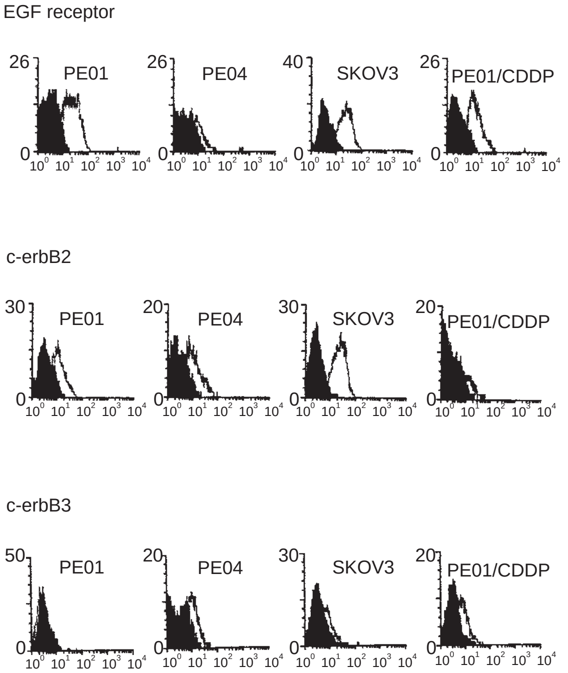

**Figure 5** Flow cytometric analysis of c-erbB receptor expression in ovarian cancer cell lines. Cells were incubated with anti-erbB antibodies (EGF receptor, clone EGFR1; c-erbB2, clone CB11; anti-erbB3, clone RTJ1) followed by sheep anti-mouse fluorescein isothiocyanate. Cells were then analysed on a FACscan. Representative profiles are shown phosphorylation of both the EGF receptor and c-erbB2 in all four cell lines consistent with ligand activation via the EGF receptor and heterodimerization with c-erbB2 (Figure 4). At concentrations of 0.3, 1 and 3 μM, the EGF receptor phosphorylation was completely blocked. The c-erbB2 phosphorylation was also decreased, but to a lesser degree in all four lines (Figure 4).

In the absence of TGF-α, only c-erbB2 phosphorylation was observed in untreated cells (Figure 4). This was also decreased after addition of ZM 252868, but only at higher concentrations of inhibitor.

### Epidermal growth factor receptor tyrosine kinase inhibitory activity of ZM 252868

The EGF receptor tyrosine kinase enzyme activity was investigated by an assay which measures the transfer of the γ-phosphate of adenosine 5′ triphosphate to the tyrosine group on a peptide which is specific for EGF receptor tyrosine kinase (Amersham). EGF, which is known to be equipotent with TGF-α in these systems, was used to activate the receptor. Addition of EGF resulted in a 22% increase in phosphorylation in PE01 cells, a 41% increase in phosphorylation in PE01$^{CDDP}$ cells and a 49% increase in phosphorylation in SKOV-3 cells (Table 1). For PE04 cells, a 9% increase in phosphorylation was observed, but this did not achieve statistical significance. These increases were completely reversed by the addition of ZM 252868 (3 μM) (Table 1). In a subsequent experiment with PE01 cells, the 33% increase in phosphorylation produced by EGF was blocked by ZM 252868 at concentrations of 0.03, 0.3 and 3 μM (Table 2). The basal level of EGF receptor activity was lower in this experiment, reflecting an unknown variable, however intra-assay changes in activity after addition of ligand and blockade by inhibitor were reproducible.

*© Cancer Research Campaign 1999*

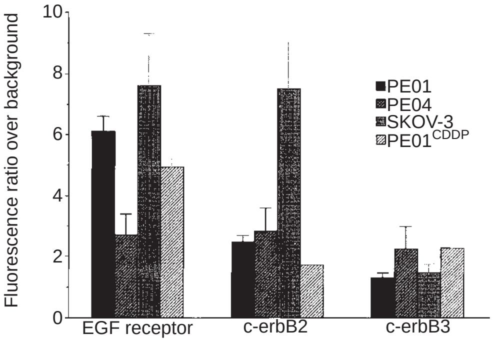

**Figure 6** c-ErbB receptor expression in ovarian cancer cell lines. Cells were incubated with anti-erbB antibodies (EGF receptor, clone EGFR1; c-erbB2, clone CB11; anti-erbB3, clone RTJ1) followed by sheep anti-mouse fluorescein isothiocyanate. Cells were then analysed on a FACscan. The ratio of median fluorescence in the presence of antibody compared with that obtained in the absence of antibody was determined. The means of at least three independent experiments ± standard deviation are shown

### Expression of erbB receptors in ovarian cancer cells

Because the activated EGF receptor has the potential to heterodimerize with other members of the c-erbB family, it was of interest to know the relative levels of expression of these proteins in the cell lines under study. The relative expression of EGF receptors, c-erbB2 and c-erbB3 in PE01, PE04, SKOV-3 and $PE01^{CDDP}$ was measured by immunofluorescence on a FACscan, and representative profiles are shown in Figure 5 and mean values obtained from three independent experiments are shown in Figure 6. The SKOV-3 cell line possessed the greatest number of EGF receptors and PE04 the least. SKOV-3 cells also had a two- to threefold greater expression of c-erbB2 than the other three line, whereas all four lines had comparable levels of c-erbB3 (Figure 6).

### DISCUSSION

ZM 252868 (also known as PD 153035) is a potent inhibitor of the EGF receptor, with an $IC_{50}$ value of 29 pM against the purified tyrosine kinase in A431 cells (Fry et al, 1994). Against other human tumour cell lines, including breast, prostatic, cervical and colon cancer cell lines, the compound has been reported to inhibit growth at concentrations ≥ 75 nM (Bos et al, 1996; Jones et al, 1997), and this is similar to the observations in these ovarian cancer cell lines wherein 100 nM inhibited the TGF-α-stimulated growth of PE01, PE04 and SKOV-3 cells. All three ovarian cancer lines have comparable levels of EGF receptor and ZM 252868 showed a similar degree of potency, with complete inhibition of growth being obtained at concentrations between 0.1 and 0.3 μM. At these concentrations, ZM 252868 inhibited TGF-α-activated tyrosine phosphorylation at the EGF receptor and partially inhibited phosphorylation at c-erbB2 consistent with its proposed mechanism of action. Because the activated EGF receptor interacts with and transphosphorylates the c-erbB2 receptor, it is not surprising that phosphorylation levels are reduced at this protein as well as at the EGF receptor, though direct effects by ZM 252868 on c-erbB2 cannot be ruled out and this is in line with previous studies (Bos et al, 1996). Although we did not investigate the specificity of ZM 252868 for the EGF receptor in this study, previous investigations have demonstrated its ability to specifically block TGF-α or EGF-stimulated growth as opposed to platelet-derived growth factor or insulin-like growth factor-stimulated growth (Fry et al, 1994*b*; Jones et al, 1997). Data obtained from the EGF receptor tyrosine kinase activity assay confirmed that inhibition of kinase activity occurred at growth inhibitory concentrations in PE01 cells. Although it cannot always be assumed that EGF receptor activation necessarily leads to a mitogenic response, the data obtained in the PE01, PE04 and SKOV-3 lines suggests that it can be and that the inhibitor blocks this action. However, the data obtained using the $PE01^{CDDP}$ model indicate that TGF-α activation may also be associated with growth inhibitory effects. TGF-α treatment produced growth inhibition in this cell line (Simpson et al, 1998), and ZM 252868 was able to antagonize this growth effect with associated reduction in EGF receptor phosphotyrosine and kinase activity. This cell line was derived from the PE01 cell line after exposure to cisplatin (Beattie et al, 1993) and, as demonstrated here, the changed growth response is not simply a result of altered erbB receptor numbers. We are currently investigating downstream responses that can be specifically associated with either growth stimulation or growth inhibition because these may help indicate the type of growth response elicited by TGF-α.

In the absence of TGF-α, ZM 252868 inhibited the growth of PE01, PE04 and SKOV-3 cells and stimulated growth of $PE01^{CDDP}$ cells compared with no treatment. This would be consistent with inhibition of the action of autocrine production of TGF-α or other EGF receptor-activating ligands which are stimulating the EGF receptor in the absence of added factors. Evidence to support such a process has been obtained from other ovarian cancer models, wherein antibodies to the EGF receptor or to TGF-α have been shown to block growth consistent with TGF-α/EGF receptor autocrine pathways being functional (Morishige et al, 1991).

Blockade of signalling via the EGF receptor appears a promising growth inhibitory strategy. Using these cell lines, we have recently investigated other approaches to neutralizing this receptor. Antibody blockade of the EGF receptor produces similar growth-reversing effects in the PE01 and $PE01^{CDDP}$ cell lines (Simpson et al, 1998), whereas antisense knockout of EGF receptor mRNA in PE01 cells also produces growth inhibition (Simpson et al, 1996). Relatively small structures such as ZM 252868 may, however, have improved pharmacokinetic properties compared with antibody or antisense delivery. Although this compound has demonstrated transient reduction of EGF receptor tyrosine phosphorylation in A431 xenografts, this was insufficient to produce growth inhibition in vivo (Kunkel et al, 1996). Closely related analogues from this class of agents with improved pharmacokinetic properties and which are active against in vivo models have now been developed (Woodburn et al, 1996, 1997), and an analogue is currently undergoing phase I studies in the UK. These data are the first to report that inhibitors of this class could have activity in ovarian cancer systems, and would indicate that this disease would be a suitable system for clinical investigations. It will be important to define tumours in the clinical setting whose growth is dependent on the EGF receptor and which are being driven by activating ligands such as TGF-α and EGF. Tyrosine phosphorylation of the EGF receptor in clinical specimens of ovarian cancer can be readily identified, and it seems likely that those tumours in which activation is found represent the target population for this inhibitor.

### ACKNOWLEDGEMENT

We are grateful to Dr Alan Wakeling, Zeneca Pharmaceuticals, Macclesfield for supplies of ZM252868.

 © Cancer Research Campaign 1999

### REFERENCES

Bartlett JMS, Langdon SP, Simpson BJB, Stewart M, Katsaros D, Sismondi P, Love S, Scott WN, Williams ARW, Lessells AM, Macleod KG, Smyth JF and Miller WR (1996) The prognostic value of epidermal growth factor receptor mRNA expression in primary ovarian cancer. *Br J Cancer* **73**: 301–306

Battaglia F, Scambia G, Benedetti Panici P, Baiocchi G, Peronne I, Iacobelli S and Mancuso S (1989) Epidermal growth factor expression in gynecological malignancies. *Gynecol Obstet Invest* **37**: 855–862

Bauknecht T, Runge M, Schwall M and Pfleiderer A (1988) Occurrence of epidermal growth factor receptors in human adnexal tumours and their prognostic value in advanced ovarian carcinomas. *Gynecol Oncol* **29**: 147–157

Bauknecht T, Angel P, Kohler M, Komoss F, Birmelin G, Pfleiderer A and Wagner E (1993) Gene structure and expression analysis of the epidermal growth factor receptor, transforming growth factor alpha, *myc*, *jun* and metallothionein in human ovarian carcinomas: classification of malignant phenotypes. *Cancer* **71**: 419–429

Beattie GJ, French RC, McGowan A, Renninson J, Meikle I and Smyth JF (1993). Cytosolic thiols in cis-platinum (cDDP) resistance in ovarian carcinoma cell lines. *Br J Cancer* **67** (suppl. XX): 130

Berchuck A, Rodriguez GC, Kamel A, Dodge RK, Soper JT, Clarke-Pearson DL and Bast RC (1991). Epidermal growth factor-receptor expression in normal ovarian epithelium and ovarian cancer. I. Correlation of receptor expression with prognostic factors in patients with ovarian cancer. *Am J Obstet Gynecol* **164**: 669–674

Bos MMEM, Mendelsohn J, Kim YM, Fry DW and Baselga J (1996) A tyrosine kinase inhibitor prevents ligand-induced receptor activation and inhibits growth of cancer cell lines expressing the epidermal growth factor receptor. *Proc Am Assoc Cancer Res* **37**: 305

Bradford M (1976) A rapid and sensitive method for the quantitation of microgram quantities of proteins utilising the principle of protein-dye binding. *Anal Biochem* **72**: 248–254

Brotherick I, Shenton BK, Angus B, Waite IS, Horne CH and Lennard TW (1995) A flow cytometric study of c-erbB-3 expression in breast cancer. *Cancer Immunol Immunother* **41**: 280–286

Carpenter G (1987) Receptors for epidermal growth factor and other polypeptide mitogens. *Ann Rev Biochem* **56**: 881–914

Chen WS, Lazar CS, Poenie M, Tsien RJ, Gill GN and Rosenfeld MG (1987). Requirements for intrinsic protein tyrosine kinase in the immediate and late actions of the EGF receptor. *Nature* **328**: 820–823

Crew AJ, Langdon SP, Miller EP and Miller WR (1992) Mitogenic effects of epidermal growth factor and transforming growth factor-α on EGF-receptor positive human ovarian cancer cell lines. *Eur J Cancer* **28**: 337–341

Egan SE and Weinberg RA (1993) The pathway of signal achievement. *Nature* **365**: 781–782

Foekens JA, van Putten WL, Portengen H, Rodenburg CJ, Reubi JC, Berns PM, Henzen-Logmans SC, van der Burg ME, Alexieva-Figush J and Klijn JG (1990). Prognostic value of pS2 protein and receptors for epidermal growth factor (EGF-R), insulin-like growth factor 1 (IGF-1-R) and somatostatin in patients with breast and ovarian cancer. *J Steroid Biochem Mol Biol* **37**: 815–821

Fry DW, Kraker AJ, Conners RC, Elliott WL, Nelson JM, Showalter HDH and Leopold WR (1994*a*) Strategies for the discovery of novel tyrosine kinase inhibitors with anticancer activity. *Anticancer Drug Dev* **9**: 331–351

Fry DW, Kraker AJ, McMichael A, Ambroso LA, Nelson JM, Leopold WR, Connors RW and Bridges AJ (1994*b*) A specific inhibitor of the epidermal growth factor receptor tyrosine kinase. *Science* **265**: 1093–1095

Graziani Y, Chayoth R, Karny N, Feldman B and Levy J (1981) Regulation of protein kinase activity by quercetin in Ehrlich ascites tumor cells. *Biochem Biophys Acta* **714**: 415–421

Henzen-Logmans SC, Berns EMJJ, Klijn JGM, Van Der Burg MEL and Foekens JA (1992) Epidermal growth factor receptor in ovarian tumours: correlation of immunohistochemistry with ligand binding assay. *Br J Cancer* **66**: 1015–1021

Honegger AM, Dull TJ, Felder S, van Obberghen E, Bellot F, Szapary D, Schmidt A, Ullrich A and Schlessinger J (1987) Point mutation at the ATP binding site of the EGF receptor abolishes protein tyrosine kinase activity and alters cellular routing. *Cell* **51**: 199–209

Jones HE, Dutkowski CM, Barrow D, Harper ME, Wakeling AE and Nicholson RI (1997) New EGF-R selective tyrosine kinase inhibitor reveals variable growth responses in prostate carcinoma cell lines PC-3 and DU-145. *Int J Cancer* **71**: 1010–1018

Kunkel MW, Hook KE, Howard CT, Przybranowski S, Robert BJ, Elliot WL and Leopold WR (1996) Inhibition of the epidermal growth factor tyrosine kinase by PD 153035 in human A431 tumors in nude mice. *Invest New Drugs* **13**: 295–302

Langdon SP, Lawrie SS, Hay FG, Hawkes MM, Mcdonald A, Hayward IP, Schol DJ, Leonard RCF and Smyth JF (1988) Characterization and properties of nine human ovarian adenocarcinoma cell lines. *Cancer Res* **48**: 6166–6172

Levitzki A and Gazit A (1995) Tyrosine kinase inhibition: an approach to drug development. *Science* **267**: 1782–1788

Morishige K, Kurachi H, Amemiya K, Fujita Y, Yamamoto T, Miyake A and Tanizawa O (1991) Evidence for the involvement of transforming growth factor α and epidermal growth factor receptor autocrine growth mechanism in primary human ovarian cancers in vitro. *Cancer Res* **51**: 5322–5328

Owens OJ, Stewart C, Brown I and Leake RE (1991) Epidermal growth factor receptors (EGFR) in human ovarian cancer. *Br J Cancer* **64**: 907–910

Rodriguez GC, Berchuck A, Whitaker RS, Schlossman D, Clarke-Pearson DL and Bast RC (1991) Epidermal growth factor receptor expression in normal ovarian epithelium and ovarian cancer. 2. Relationship between receptor expression and response to epidermal growth factor. *Am J Obstet Gynecol* **164**: 745–750

Scambia G, Benedetti-Panici P, Battaglia F, Ferrandina G, Gaggini C and Mancuso S (1991) Presence of epidermal growth factor (EGF) receptor and proliferative response to EGF in six human carcinoma cell lines. *Int J Gynecol Cancer* **1**: 253–258

Scambia G, Benedetti-Panici P, Battaglia F, Ferrandina G, Baiocchi G, Greggi S, de Vincenzo R and Mancuso S (1992) Significance of epidermal growth factor receptor in advanced ovarian cancer. *J Clin Oncol* **10**: 529–535

Simpson BJB, Phillips HA, Lessells AM, Langdon SP and Miller WR (1995) c-ErbB growth factor receptor proteins in ovarian tumours. *Int J Cancer* **64**: 202–206

Simpson BJB, Macleod KG, Miller WR and Langdon SP (1996) Antisense oligonucleotide to epidermal growth factor receptor in ovarian cancer. *Br J Cancer* **73** (suppl. XXVI): 62

Simpson BJB, Langdon SP, Rabiasz GJ, Macleod KG, Hirst GL, Bartlett JMS, Crew AJ, Hawkins RA, Macineira-Perez PP, Smyth JF and Miller WR (1998) Estrogen regulation of transforming growth factor-α in ovarian cancer. *J Steroid Biochem Mol Biol* **64**: 137–145

Wakeling AE, Barker AJ, Davies DH, Brown DS, Green LR, Cartlidge SA and Woodburn JR (1996) Specific inhibition of epidermal growth factor receptor tyrosine kinase by 4-anilinoquinazolines. *Breast Cancer Res Treat* **38**: 67–73

Woodburn JR, Barker AJ, Wakeling AE, Valcaccia BE, Cartlidge SA and Davies DH (1996) 6-Amino-4-(3-methylphenylamino)-quinazoline: an EGF receptor tyrosine kinase inhibitor with activity in a range of human tumour xenografts. *Proc Am Assoc Cancer Res* **37**: 2665

Woodburn JR, Barker AJ, Gibson KH, Ashton SE, Wakeling AE, Curry BJ, Scarlett L and Henthorn LR (1997). ZD1839, an epidermal growth factor tyrosine kinase inhibitor selected for clinical development. *Proc Am Assoc Cancer Res* **38**: 4251

Zhou L and Leung BS (1992) Growth regulation of ovarian cancer cells by epidermal growth factor and transforming growth factor α and β1. *Biochim Biophys Acta* **1180**: 130–136

© Cancer Research Campaign 1999
